# Supplementary material for: Science through Wikipedia: A novel representation of open knowledge through co-citation networks
Source: PLoS One. 2020 Feb 10;15(2):e0228713. doi: 10.1371/journal.pone.0228713 (PMC7010282; doi:10.1371/journal.pone.0228713)
Supplement: S1 Table — (PDF) [file pone.0228713.s001.pdf]

Descriptive statistics of references made by Wikipedia entries and citations that scientific articles receive from Wikipedia entries by main fields

	Descriptive statistics of references made by Wikipedia entries to articles by main field					Descriptive statistics of citations that scientific articles receive from Wikipedia entries by main fields				
**Main field**	**Wikipedia entries**	**References (% total)**	**Mean (± standard deviation)**	**Median**	**Range**	**Articles**	**References (% total)**	**Mean (±standard deviation)**	**Median**	**Range**
Agricultural and Biological Sciences	58 489	126 377 (14.91%)	2.16 (± 3.85)	1	241	80 786	126 377 (14.91%)	1.56 (± 5.67)	1	927
Arts and Humanities	28 223	46 487 (5.49%)	1.65 (± 1.80)	1	55	36 199	46 487 (5.49%)	1.28 (± 1.19)	1	107
Biochemistry, Genetics and Molecular Biology	64 945	266 956 (31.50%)	4.11 (± 6.10)	2	185	181 954	266 956 (31.50%)	1.47 (± 11.77)	1	3591
Business, Management and Accounting	4127	7416 (0.88%)	1.80 (± 2.36)	1	71	6325	7416 (0.88%)	1.17 (± 0.62)	1	15
Chemical Engineering	9397	17 791 (2.10%)	1.89 (± 2.37)	1	38	14 002	17 791 (2.10%)	1.27 (± 2.88)	1	205
Chemistry	17 381	43 262 (5.10%)	2.49 (± 3.80)	1	101	34 889	43 262 (5.10%)	1.24 (± 1.20)	1	124
Computer Science	9286	16 874 (1.99%)	1.82 (± 2.13)	1	60	13 631	16 874 (1.99%)	1.24 (± 0.75)	1	15
Decision Sciences	2605	4159 (0.49%)	1.60 (± 1.63)	1	29	3420	4159 (0.49%)	1.22 (± 0.65)	1	14
Dentistry	992	2407 (0.28%)	2.43 (± 3.50)	1	43	2116	2407 (0.28%)	1.14 (± 0.52)	1	7
Earth and Planetary Sciences	24 663	63 525 (7.50%)	2.58 (± 5.05)	1	264	33 875	63 525 (7.50%)	1.88 (± 14.70)	1	1483
Economics, Econometrics and Finance	4818	9915 (1.17%)	2.06 (± 3.06)	1	89	7775	9915 (1.17%)	1.28 (± 1.46)	1	107
Energy	2122	3615 (0.43%)	1.70 (± 1.98)	1	28	2957	3615 (0.43%)	1.22 (± 0.82)	1	18
Engineering	11 135	19 027 (2.25%)	1.71 (± 2.13)	1	42	14 940	19 027 (2.25%)	1.27 (± 2.78)	1	205
Environmental Science	17 516	30 653 (3.62%)	1.75 (± 2.11)	1	67	22 990	30 653 (3.62%)	1.33 (± 1.70)	1	158
Health Professions	3177	5762 (0.68%)	1.81 (± 2.29)	1	46	4882	5762 (0.68%)	1.18 (± 0.58)	1	13

Immunology and Microbiology	24 824	54 128 (6.39%)	2.18 (± 2.99)	1	80	39 890	54 128 (6.39%)	1.36 (± 3.05)	1	460
Materials Science	5783	13 253 (1.56%)	2.29 (± 3.45)	1	60	11 342	13 253 (1.56%)	1.17 (± 0.57)	1	11
Mathematics	10 891	20 672 (2.44%)	1.90 (± 2.43)	1	67	16 458	20 672 (2.44%)	1.26 (± 3.66)	1	460
Medicine	72 384	276 125 (32.58%)	3.81 (± 6.83)	2	221	206 576	276 125 (32.58%)	1.34 (± 9.48)	1	3591
Multidisciplinary	33 943	72 346 (8.54%)	2.13 (± 2.72)	1	99	38 422	72 346 (8.54%)	1.88 (± 26.40)	1	4997
Neuroscience	15 588	41 771 (4.93%)	2.68 (± 4.05)	1	89	32 760	41 771 (4.93%)	1.28 (± 1.06)	1	61
Nursing	4855	8856 (1.04%)	1.82 (± 2.14)	1	31	7232	8856 (1.04%)	1.22 (± 0.78)	1	33
Pharmacology, Toxicology and Pharmaceutics	14 706	33 758 (3.98%)	2.30 (± 3.00)	1	75	26 578	33 758 (3.98%)	1.27 (± 1.00)	1	55
Physics and Astronomy	21 829	64 001 (7.55%)	2.93 (± 4.42)	1	115	37 768	64 001 (7.55%)	1.69 (± 13.90)	1	1483
Psychology	9235	34 051 (4.02%)	3.69 (± 6.24)	1	109	27 033	34 051 (4.02%)	1.26 (± 0.94)	1	87
Social Sciences	33 104	61 986 (7.31%)	1.87 (± 2.40)	1	56	48 877	61 986 (7.31%)	1.27 (± 0.96)	1	84
Veterinary	2314	4403 (0.52%)	1.90 (± 2.47)	1	56	3719	4403 (0.52%)	1.18 (± 0.66)	1	16
