# Supplementary material for: Science through Wikipedia: A novel representation of open knowledge through co-citation networks
Source: PLoS One. 2020 Feb 10;15(2):e0228713. doi: 10.1371/journal.pone.0228713 (PMC7010282; doi:10.1371/journal.pone.0228713)
Supplement: S2 Table — (PDF) [file pone.0228713.s002.pdf]

Most cited articles in Wikipedia

	Article	Main fields	Wikipedia citations	Scopus citations	Difference
1	Mammalian Gene Collection (MGC) Program Team. (2002). Generation and initial analysis of more than 15,000 full-length human and mouse cDNA sequences. *Proceedings of the National Academy of Sciences*, *99*(26), 16899-16903.	Biology / Genetics	4997	1288	3709
2	Gerhard, D. S., Wagner, L., Feingold, E. A., Shenmen, C. M., Grouse, L. H., Schuler, G., ... & Guyer, M. (2004). The status, quality, and expansion of the NIH full-length cDNA project: the Mammalian Gene Collection (MGC). *Genome research*, *14*(10B), 2121-2127.	Biology / Genetics	3591	341	3250
3	Ota, T., Suzuki, Y., Nishikawa, T., Otsuki, T., Sugiyama, T., Irie, R., ... & Kimura, K. (2004). Complete sequencing and characterization of 21,243 full-length human cDNAs. *Nature genetics*, *36*(1), 40.	Biology / Genetics	2346	529	1817
4	Van Leeuwen, F. (2007). Validation of the new Hipparcos reduction. *Astronomy & Astrophysics*, *474*(2), 653-664.	Astronomy	1483	2055	-572
5	Menzel, D. H., Minnaert, M., Levin, B., Dollfus, A., & Bell, B. (1971). Report on lunar nomenclature by the working group of Commission 17 of the IAU. *Space Science Reviews*, *12*(2), 136-186.	Astronomy	1450	6	1444
6	Kazuo, M., & Sumio, S. (1994). Oligo-capping: a simple method to replace the cap structure of eukaryotic mRNAs with oligoribonucleotides. *Gene*, *138*(1-2), 171-174.	Health Sciences / Microbiology	1019	486	533
7	Rual, J. F., Venkatesan, K., Hao, T., Hirozane-Kishikawa, T., Dricot, A., Li, N., ... & Klitgord, N. (2005). Towards a proteome-scale map of the human protein–protein interaction network. Nature, 437(7062), 1173.	Biology / Biochemistry	986	1783	-797
8	Suzuki, Y., Yoshitomo-Nakagawa, K., Maruyama, K., Suyama, A., & Sugano, S. (1997). Construction and characterization of a full length-enriched and a 5′-end-enriched cDNA library. *Gene*, *200*(1-2), 149-156.	Biology / Genetics	983	223	760

9	Sihvonen, P. (2005). Phylogeny and classification of the Scopulini moths (Lepidoptera: Geometridae, Sterrhinae). *Zoological Journal of the Linnean Society*, *143*(4), 473-530.	Biology / Genetics	927	25	902
10	Vereš, P., Jedicke, R., Fitzsimmons, A., Denneau, L., Granvik, M., Bolin, B., ... & Flewelling, H. (2015). Absolute magnitudes and slope parameters for 250,000 asteroids observed by Pan-STARRS PS1–Preliminary results. *Icarus*, *261*, 34-47.	Information Science and Computational Science / AI	923	21	902
11	Mainzer, A., Grav, T., Masiero, J., Hand, E., Bauer, J., Tholen, D., ... & Watkins, J. (2011). NEOWISE studies of spectrophotometrically classified asteroids: Preliminary results. *The Astrophysical Journal*, *741*(2), 90.	Information Science and Computational Science / AI	874	56	818
12	Welch, J. J. (2010). The “Island Rule” and deep-sea gastropods: Re-examining the evidence. *PloS one*, *5*(1), e8776.	Natural Science and Biology	654	9	645
13	Olsen, J. V., Blagoev, B., Gnad, F., Macek, B., Kumar, C., Mortensen, P., & Mann, M. (2006). Global, in vivo, and site-specific phosphorylation dynamics in signaling networks. *Cell*, *127*(3), 635-648.	Biology / Biochemistry	622	2232	-1610
14	Kimura, K., Wakamatsu, A., Suzuki, Y., Ota, T., Nishikawa, T., Yamashita, R., ... & Ishii, S. (2006). Diversification of transcriptional modulation: large-scale identification and characterization of putative alternative promoters of human genes. *Genome research*, *16*(1), 55-65.	Biology / Genetics	585	289	296
15	Masiero, J. R., Mainzer, A. K., Grav, T., Bauer, J. M., Cutri, R. M., Nugent, C., & Cabrera, M. S. (2012). Preliminary analysis of WISE/NEOWISE 3-band cryogenic and post-cryogenic observations of main belt asteroids. *The Astrophysical Journal Letters*, *759*(1), L8.	Physics	533	32	501
16	Bonaldo, M. D. F., Lennon, G., & Soares, M. B. (1996). Normalization and subtraction: two approaches to facilitate gene discovery. *Genome research*, *6*(9), 791-806.	Biology / Genetics	518	442	76
17	Hartley, J. L., Temple, G. F., & Brasch, M. A. (2000). DNA cloning using in vitro site-specific recombination. *Genome research*, *10*(11), 1788-1795.	Biology / Genetics	507	642	-135

18	Masiero, J. R., Grav, T., Mainzer, A. K., Nugent, C. R., Bauer, J. M., Stevenson, R., & Sonnett, S. (2014). Main-belt asteroids with WISE/NEOWISE: Near-infrared albedos. *The Astrophysical Journal*, *791*(2), 121.	Physics	501	27	474
19	Nielsen, E. S., Robinson, G. S., & Wagner, D. L. (2000). Ghost-moths of the world: a global inventory and bibliography of the Exoporia (Mnesarchaeoidea and Hepialoidea)(Lepidoptera). *Journal of Natural History*, *34*(6), 823-878.	Biology / Ecology	491	41	450
20	Wiemann, S., Weil, B., Wellenreuther, R., Gassenhuber, J., Glassl, S., Ansorge, W., ... & Lauber, J. (2001). Toward a catalog of human genes and proteins: sequencing and analysis of 500 novel complete protein coding human cDNAs. *Genome Research*, *11*(3), 422-435.	Biology / Biochemistry	472	155	317
