# Supplementary material for: Science through Wikipedia: A novel representation of open knowledge through co-citation networks
Source: PLoS One. 2020 Feb 10;15(2):e0228713. doi: 10.1371/journal.pone.0228713 (PMC7010282; doi:10.1371/journal.pone.0228713)
Supplement: S3 Table — (PDF) [file pone.0228713.s003.pdf]

Most cited journals in Wikipedia

	Journal	Articles	Citations	References/ articles	Open Access	Area
1	Nature	13 312	26 434	1.986	No	Multidisciplinary
2	Proceedings of the National Academy of Sciences of the United States of America	12 234	24 104	1.970	No	Multidisciplinary
3	Journal of Biological Chemistry	16 611	21 921	1.320	No	Life Sciences
4	Science	10 191	17 853	1.752	No	Multidisciplinary
5	PLoS ONE	5793	9603	1.658	Yes	Health Sciences/Life Sciences
6	Genome Research	613	8839	1.419	No	Health Sciences/Life Sciences
7	Astrophysical Journal	3722	8552	2.298	No	Physical Sciences
8	Cell	3673	7223	1.967	No	Life Sciences
9	Astronomy and Astrophysics	1584	5546	3.501	No	Physical Sciences
10	Journal of the American Chemical Society	4549	5529	1.215	No	Life Sciences/Physical Sciences
11	Gene	1848	5468	2.959	No	Health Sciences/Life Sciences
12	New England Journal of Medicine	3628	5045	1.391	No	Health Sciences
13	Nature Genetics	1618	4853	2.999	No	Life Sciences
14	Genomics	2766	4685	1.694	No	Life Sciences
15	The Lancet	3000	4634	1.545	No	Health Sciences
16	Nucleic Acids Research	2928	4570	1.561	Yes	Life Sciences
17	Biochemical and Biophysical Research Communications	3584	4425	1.235	No	Life Sciences
18	Physical Review Letters	3037	4262	1403	No	Physical Sciences
19	International Journal of Systematic and Evolutionary Microbiology	2791	4120	1,476	No	Health Sciences/Life Sciences
20	Biochemistry	3307	3886	1.175	No	Life Sciences
