# Supplementary material for: Science through Wikipedia: A novel representation of open knowledge through co-citation networks
Source: PLoS One. 2020 Feb 10;15(2):e0228713. doi: 10.1371/journal.pone.0228713 (PMC7010282; doi:10.1371/journal.pone.0228713)
Supplement: S4 Table — (PDF) [file pone.0228713.s004.pdf]

Main journals by measures of centrality in full co-citation network, co-citation PFNET and filtered co-citation PFNET

	Degree	Betweenness	Closeness	Eigenvector
**Full co-citation network**				
1	Science (7119)	Science (0.093)	Science (0.671)	Science (1.0)
2	Nature (7052)	Nature (0.092)	Nature (0.668)	Proceedings of the National Academy of Sciences of the United States of America (0.998)
3	Proceedings of the National Academy of Sciences of the United States of America (6901)	Proceedings of the National Academy of Sciences of the United States of America (0.079)	Proceedings of the National Academy of Sciences of the United States of America (0.662)	Nature (0.996)
4	PLoS ONE (6561)	PLoS ONE (0.068)	PLoS ONE (0.650)	PLoS ONE (0.982)
5	The Lancet (5024)	The Lancet (0.031)	The Lancet (0.598)	New England Journal of Medicine (0.883)
6	New England Journal of Medicine (4991)	New England Journal of Medicine (0.028)	New England Journal of Medicine (0.595)	The Lancet (0.874)
7	JAMA - Journal of the American Medical Association (4200)	JAMA - Journal of the American Medical Association (0.018)	JAMA - Journal of the American Medical Association (0.573)	JAMA - Journal of the American Medical Association (0.798)
8	Scientific Reports (3778)	Scientific Reports (0.015)	Scientific Reports (0.563)	Journal of Biological Chemistry (0.761)
9	Journal of Biological Chemistry (3574)	Lecture Notes in Computer Science (0.010)	Journal of Biological Chemistry (0.549)	Scientific Reports (0.727)
10	Cochrane Database of Systematic Reviews (3410)	Scientific American (0.010)	Annals of the New York Academy of Sciences (0.545)	Cell (0.723)
**Co-citation PFNET**				
1	Proceedings of the National Academy of Sciences of the United States of America (1604)	Proceedings of the National Academy of Sciences of the United States of America (0.198)	Proceedings of the National Academy of Sciences of the United States of America (0.433)	Proceedings of the National Academy of Sciences of the United States of America (1.0)
2	Nature (1431)	Nature (0.176)	Nature (0.426)	Nature (0.883)

3	Science (1253)	Science (0.150)	Science (0.417)	Science (0.774)
4	PLoS ONE (911)	PLoS ONE (0.094)	PLoS ONE (0.398)	PLoS ONE (0.555)
5	New England Journal of Medicine (844)	New England Journal of Medicine (0.074)	Journal of Hydroinformatics (0.388)	New England Journal of Medicine (0.472)
6	The Lancet (716)	The Lancet (0.056)	New England Journal of Medicine (0.385)	The Lancet (0.396)
7	Journal of Biological Chemistry (393)	JAMA - Journal of the American Medical Association (0.020)	Materials Research Letters (0.384)	JAMA - Journal of the American Medical Association (0.213)
8	JAMA - Journal of the American Medical Association (382)	Journal of Biological Chemistry (0.019)	Open Neurology Journal (0.372)	Journal of Biological Chemistry (0.201)
9	Cochrane Database of Systematic Reviews (306)	Physical Review Letters (0.015)	The Lancet (0.372)	Cochrane Database of Systematic Reviews (0.167)
10	Physical Review Letters (248)	Journal of the American Chemical Society (0.0145)	Journal of Sensory Studies (0.370)	Journal of Hydroinformatics (0.167)
**Filtered co-citation PFNET**				
1	Proceedings of the National Academy of Sciences of the United States of America (251)	Proceedings of the National Academy of Sciences of the United States of America (0.881)	Proceedings of the National Academy of Sciences of the United States of America (0.491)	Proceedings of the National Academy of Sciences of the United States of America (1.0)
2	Nature (76)	Nature (0.389)	Nature (0.413)	Nature (0.198)
3	Journal of Biological Chemistry (41)	Science (0.220)	Cold Spring Harbor Symposia on Quantitative Biology (0.383)	Journal of Biological Chemistry (0.112)
4	Science (33)	New England Journal of Medicine (0.214)	Neuroscience and Biobehavioral Reviews (0.367)	New England Journal of Medicine (0.103)
5	New England Journal of Medicine (32)	Journal of Biological Chemistry (0.120)	Nature Structural and Molecular Biology (0.366)	Cold Spring Harbor Symposia on Quantitative Biology (0.083)
6	Journal of the American Chemical Society (26)	The Lancet (0.087)	Annual Review of Entomology (0.358)	Neuroscience and Biobehavioral Reviews (0.081)

7	Physical Review Letters (19)	Journal of the American Chemical Society (0.081)	New England Journal of Medicine (0.357)	Nature Structural and Molecular Biology (0.077)
8	The Lancet (18)	PLoS ONE (0.072)	Journal of Biological Chemistry (0.344)	PLoS ONE (0.076)
9	Journal of Personality and Social Psychology (17)	Physical Review Letters (0.063)	Journal of the National Cancer Institute (0.343)	Molecular and Cellular Neurosciences (0.070)
10	PLoS ONE (15)	Psychological Review (0.059)	PLoS ONE (0.338)	Journal of Neuroscience (0.070)
