# Supplementary material for: Science through Wikipedia: A novel representation of open knowledge through co-citation networks
Source: PLoS One. 2020 Feb 10;15(2):e0228713. doi: 10.1371/journal.pone.0228713 (PMC7010282; doi:10.1371/journal.pone.0228713)
Supplement: S5 Table — (PDF) [file pone.0228713.s005.pdf]

Descriptive statistics of references made by Wikipedia entries and citations that articles receive from Wikipedia by areas

	Descriptive statistics of references made by Wikipedia entries to articles by areas					Descriptive statistics of citations that articles receive from Wikipedia entries by areas				
**Area**	**Wikipedia entries**	**References (% total)**	**Mean (±standard deviation)**	**Median**	**Range**	**Articles**	**References (% total)**	**Mean (±standard deviation)**	**Median**	**Range**
Health Sciences	73 402	282 750 (33.36%)	3.85 (± 6.89)	2	221	212 292	282 750 (33.36%)	1.33 (± 9.35)	1	3591
Life Sciences	102 933	414 400 (48.90%)	4.03 (± 6.61)	2	265	287 674	414 400 (48.90%)	1.44 (± 9.70)	1	3591
Multidisciplinary	33 943	72 346 (8.54%)	2.13 (± 2.72)	1	99	38 422	72 346 (8.54%)	1.88 (± 26.40)	1	4997
Physical Sciences	73 453	195 678 (23.09%)	2.66 (± 4.65)	1	301	137 492	195 678 (23.09%)	1.42 (± 7.54)	1	1483
Social Sciences & Humanities	49 688	115 947 (13.68%)	2.33 (± 3.96)	1	127	91 617	115 947 (13.68%)	1.27 (± 1.01)	1	107
