# Supplementary material for: Science through Wikipedia: A novel representation of open knowledge through co-citation networks
Source: PLoS One. 2020 Feb 10;15(2):e0228713. doi: 10.1371/journal.pone.0228713 (PMC7010282; doi:10.1371/journal.pone.0228713)
Supplement: S6 Table — (PDF) [file pone.0228713.s006.pdf]

Percentage of articles by main field in Scopus and Wikipedia and its differences

Main field	Scopus*	Wikipedia	Difference
Agricultural and Biological Sciences	4.64%	9.36%	4.72%
Arts and Humanities	2.69%	3.45%	0.76%
Biochemistry, Genetics and Molecular Biology	8.92%	19.78%	10.86%
Business, Management and Accounting	1.13%	0.55%	-0.58%
Chemical Engineering	2.44%	1.32%	-1.12%
Chemistry	5.96%	3.21%	-2.75%
Computer Science	2.29%	1.25%	-1.04%
Decision Sciences	0.44%	0.31%	-0.13%
Dentistry	0.42%	0.18%	-0.24%
Earth and Planetary Sciences	2.60%	4.71%	2.11%
Economics, Econometrics and Finance	0.86%	0.74%	-0.12%
Energy	1.24%	0.27%	-0.97%
Engineering	7.90%	1.41%	-6.49%
Environmental Science	2.97%	2.27%	-0.70%
Health Professions	0.77%	0.43%	-0.34%
Immunology and Microbiology	2.13%	4.01%	1.88%
Materials Science	5.03%	0.98%	-4.05%
Mathematics	2.75%	1.53%	-1.22%
Medicine	24.22%	20.46%	-3.76%
Multidisciplinary	0.99%	5.36%	4.37%
Neuroscience	1.76%	3.10%	1.34%
Nursing	1.12%	0.66%	-0.46%
Pharmacology, Toxicology and Pharmaceutics	2.93%	2.50%	-0.43%
Physics and Astronomy	6.87%	4.74%	-2.13%
Psychology	1.53%	2.52%	0.99%
Social Sciences	4.78%	4.59%	-0.19%
Veterinary	0.60%	0.33%	-0.27%
**total of 62 821 260 scientific articles indexed in the database*			
