# Supplementary material for: Science through Wikipedia: A novel representation of open knowledge through co-citation networks
Source: PLoS One. 2020 Feb 10;15(2):e0228713. doi: 10.1371/journal.pone.0228713 (PMC7010282; doi:10.1371/journal.pone.0228713)
Supplement: S7 Table — (PDF) [file pone.0228713.s007.pdf]

Top 25 most cited fields with local measures of centrality

Field	Cites	Articles	Closeness	Betweenness	Degree	Eigenvector
General Medicine	108 131	78 115	0.386	0.860	130	1000
Molecular Biology	98 118	70 485	0.334	0.492	24	0.145
Biochemistry	78 704	60 917	0.262	0.163	5	0.027
Genetics	77 920	42 313	0.251	0.006	2	0.017
Multidisciplinary	72 346	38 422	0.261	0.143	5	0.024
Ecology, Evolution, Behavior and Systematics	72 079	43 699	0.294	0.180	29	0.154
Cell Biology	65 788	48 948	0.251	0.000	1	0.015
General Biochemistry, Genetics and Molecular Biology	39 606	24 290	0.251	0.000	1	0.015
Space and Planetary Science	33 956	14 266	0.208	0.024	4	0.007
Astronomy and Astrophysics	31 704	12 657	0.172	0.000	1	0.002
General Agricultural and Biological Sciences	27 194	16 185	0.228	0.000	1	0.017
Animal Science and Zoology	25 600	15 293	0.228	0.000	1	0.017
Genetics (clinical)	24 106	11 328	0.201	0.000	1	0.003
Pharmacology	21 429	16 702	0.279	0.000	1	0.086
General Chemistry	21 037	16 595	0.215	0.146	22	0.042
Plant Science	20 398	12 282	0.228	0.006	2	0.018
Psychiatry and Mental health	18 069	14 595	0.314	0.339	14	0.113
History	17 742	13 019	0.158	0.042	8	0.016
Immunology	17 427	13 900	0.279	0.000	1	0.086
Microbiology	17 233	13 123	0.279	0.000	1	0.086
General Neuroscience	16 933	12 549	0.251	0.006	2	0.017
Sociology and Political Science	16 592	12 919	0.187	0.237	18	0.036
Clinical Neurology	15 473	12 816	0.279	0.000	1	0.086
General Physics and Astronomy	14 770	10 927	0.211	0.100	9	0.016
Physiology	14 725	10 973	0.279	0.000	1	0.086
Cancer Research	14 643	11 860	0.251	0.000	1	0.015
Infectious Diseases	13 761	10 860	0.279	0.000	1	0.086
