# Supplementary material for: Science through Wikipedia: A novel representation of open knowledge through co-citation networks
Source: PLoS One. 2020 Feb 10;15(2):e0228713. doi: 10.1371/journal.pone.0228713 (PMC7010282; doi:10.1371/journal.pone.0228713)
Supplement: S8 Table — (PDF) [file pone.0228713.s008.pdf]

Comparison of the most cited journals on Wikipedia and Altmetric.com

Journal	Wikipedia Mentions	Journal	Altmetric Mentions
Nature	34 424	Nature	6 847 973
Proceedings of the National Academy of Sciences of the United States of America	28 276	Science	2 879 553
Science	24 758	New England Journal of Medicine	1 754 603
Journal of Biological Chemistry	22 174	British Medical Journal	1 726 136
PLoS ONE	12 705	Proceedings of the National Academy of Sciences of the United States of America	1 644 974
The Astrophysical Journal	11 298	PLoS ONE	1 638 487
Zookeys	9 038	JAMA: Journal of the American Medical Association	1 261 491
Genome Research	8 894	The Lancet	1 181 206
Cell	7 768	Scientific Reports	931 194
Astronomy and Astrophysics	7 149	Nature Communications	895 397
Journal of the American Chemical Society	6 790	Cell	502 674
The Lancet	6 630	IEEE Spectrum	470 470
British Medical Journal	6 468	JAMA Internal Medicine	463 672
Nucleic Acids Research	6 027	British Journal of Sports Medicine	357 935
Physical Review Letters	5 896	Scientific American	335 845
International Journal of Systematic and Evolutionary Microbiology	5 887	Annals of Internal Medicine	305 525
New England Journal of Medicine	5 864	Science Advances	300 097
Nature Genetics	5 324	Current Biology	289 391
Biochemical & Biophysical Research Communications	5 098	Nature Medicine	269 194
The Astronomical Journal	4 932	Pediatrics	267 039
***Data retrieved from Altmetric Explorer in 4 December 2019*			
