# Supplementary figures and images for: Science through Wikipedia: A novel representation of open knowledge through co-citation networks
Source: PLoS One. 2020 Feb 10;15(2):e0228713. doi: 10.1371/journal.pone.0228713 (PMC7010282; doi:10.1371/journal.pone.0228713)

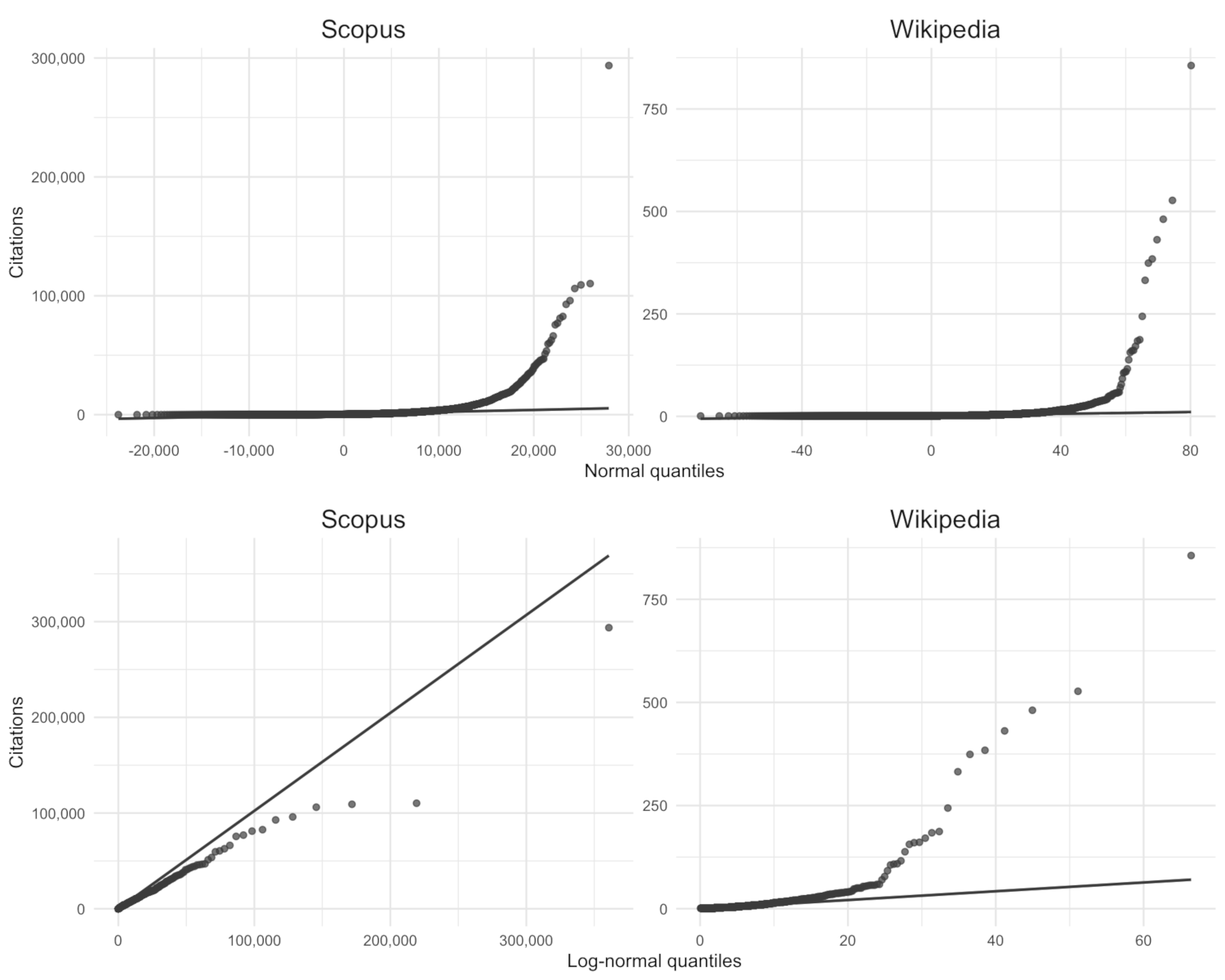

Supplement: S1 Fig — (TIFF) [file pone.0228713.s009.tiff]

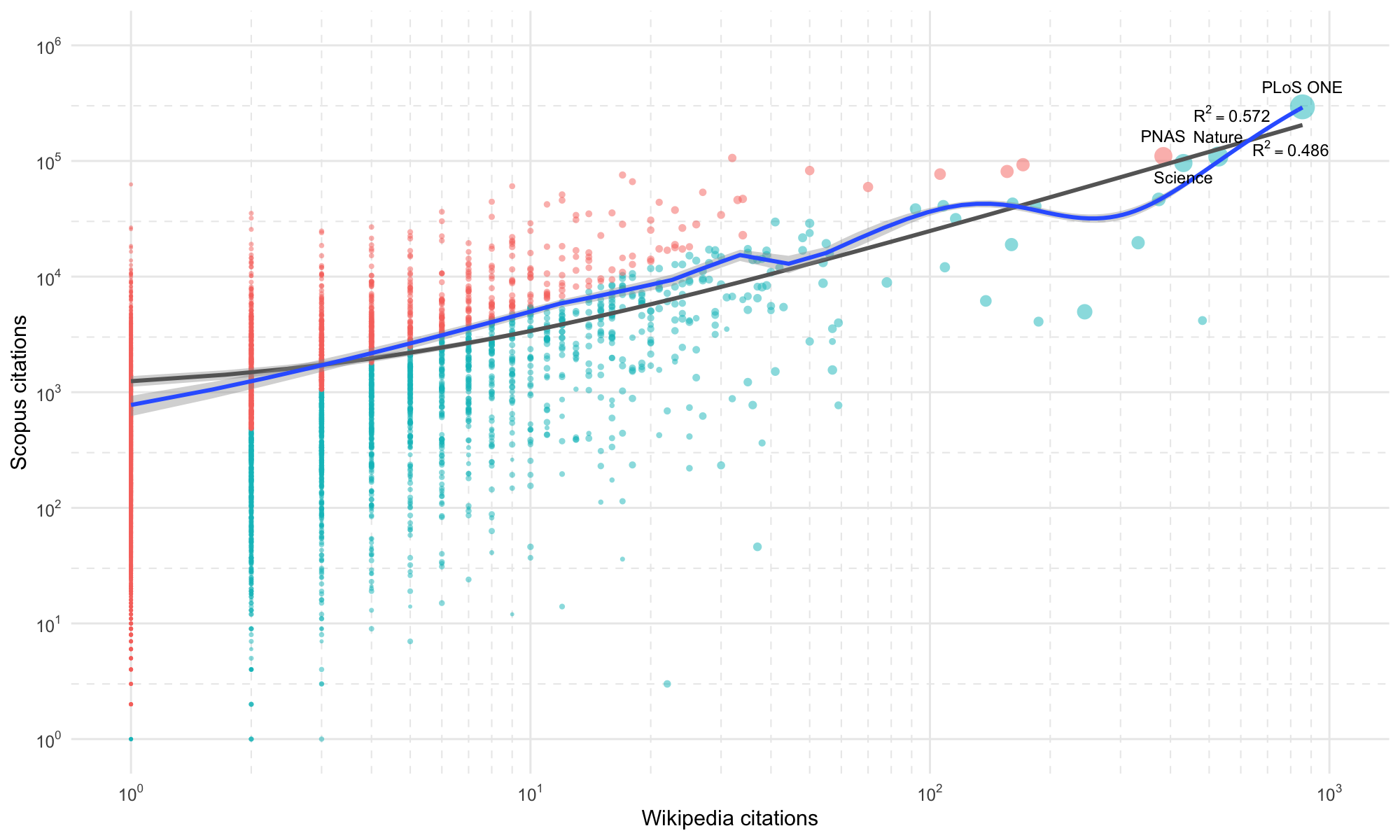

Supplement: S2 Fig — (TIFF) [file pone.0228713.s010.tiff]

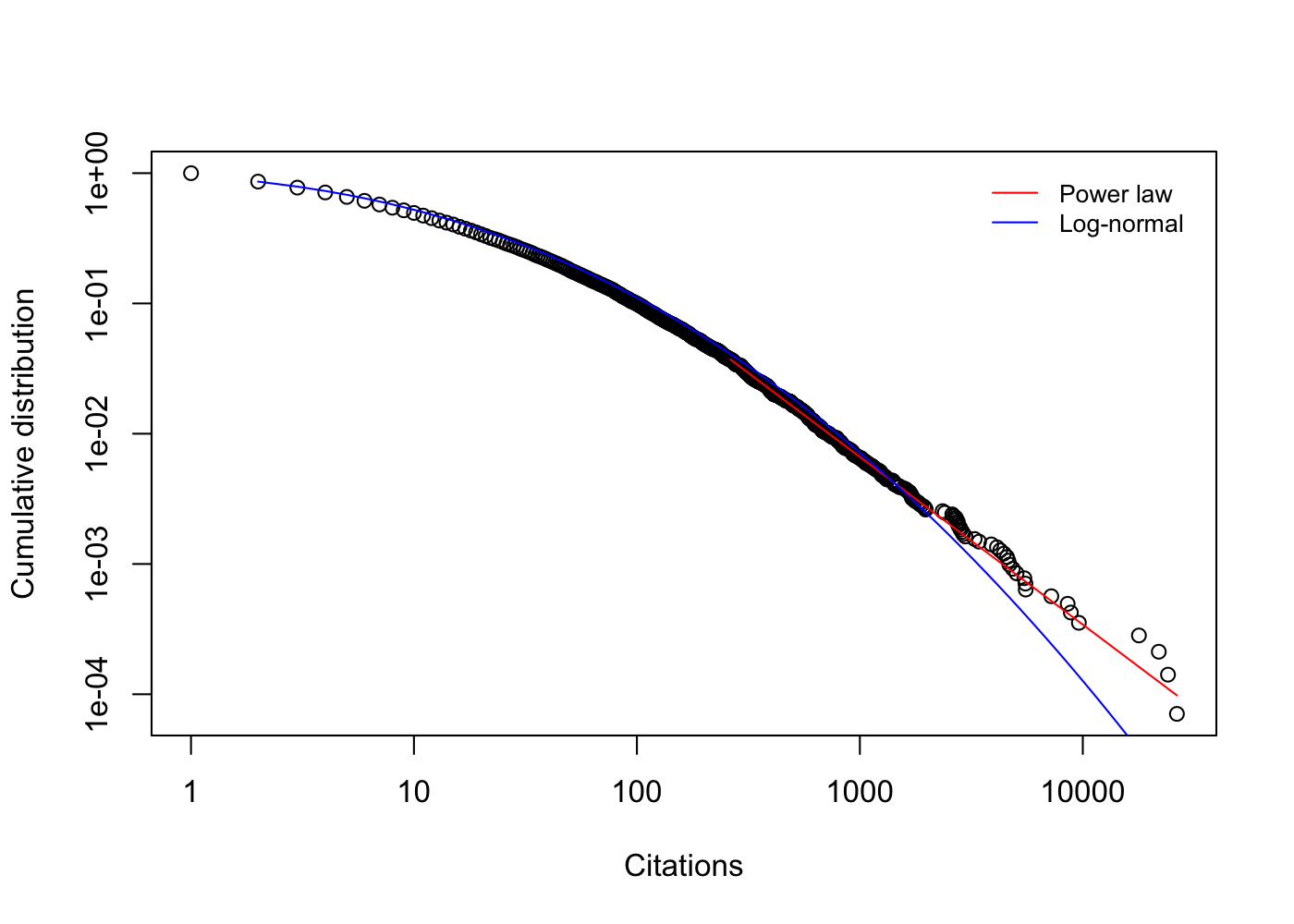

Supplement: S3 Fig — (TIFF) [file pone.0228713.s011.tiff]
