# Supplementary material for: Science through Wikipedia: A novel representation of open knowledge through co-citation networks
Source: PLoS One. 2020 Feb 10;15(2):e0228713. doi: 10.1371/journal.pone.0228713 (PMC7010282; doi:10.1371/journal.pone.0228713)
Supplement: Attachment — Submitted filename: Response.docx [file pone.0228713.s012.docx]

# Response to the revision of the manuscript: “Science through Wikipedia: a novel representation of open knowledge through co-citation networks”

We greatly appreciate the review of the paper by the two reviewers. We have revised it based on their very helpful comments. Below are highlights of our revisions and the responses to reviewers’ comments.

- In relation to the comments of reviewer 1 we have made the following changes:
  - In-depth analysis of statistics in general.
  - Comparison of Wikipedia citations with those of Scopus through two statistical models, including a new figure.
  - Analysis of the distribution of Wikipedia citations to check the existence of a power-law distribution, including a new figure as complementary material.
  - Creation of a new section in the discussion in which we compare Scopus and Wikipedia.
  - Addition of two paragraphs explaining Wikipedia's control over its editions and bots, regarding the references.
  - Comparison of the most cited journals in Wikipedia and Altmetrics, including a new table as complementary material.
- In relation to the reviewer's comments 2 we have made the following changes:
  - Addition of a new paragraph detailing the choice of co-citation maps.
  - Expansion of the explanation of the selection of the Pathfinder algorithm, including a new complete figure of Wikipedia co-citations with and without Pathfinder.
  - Inclusion of 11 new bibliographical references.

**Comments of reviewer 1**

| **Comments** | **Response** |
| --- | --- |
| The results of this paper, despite its large amount, show little significance as findings and are sometimes misleading. The authors often abruptly stop their investigation after presenting some statistics, leaving the critical and interesting questions unanswered. | We thank the reviewer for the in-depth review. Considering the criticism made, we have carried out an in-depth revision of the aspects indicated (writing, data analysis, etc.), particularly those indicated in his/her comments. Below we detail the changes made and the modifications introduced in the text for each of the criticisms made by the reviewer. |
| **Additional Analysis & Citation distributions**  For example, the paper states that each Wikipedia entry includes on average 4.373 references. However, older and more popular entries may be better developed, and therefore includes more references. It is rather confusing what information or message this average delivers to the readers, especially when the standard error is so large as well (8.351). Since the distribution is likely skewed (judging from the large stderr and what's commonly observed in citation analysis), the averages may not representative statistics after all. Is the distribution of references per entry/edit log-normal or power-law? On the per-entry level, how does this depend on the entry's age, birth year/month, popularity, topic, language, etc? On the per-edit level, how does this depend on the editors' experience, expertise area, edit year/month, language, etc? Can you show causality via matching? Are these relationships different when comparing editors on Wikipedia and researchers in academia? Stronger/weaker homophily in languages? | The comments are interesting and we have followed the main recommendations that the reviewer points out. But it should be considered that the analysis and in-depth citation patterns in Wikipedia are not the aim of the paper. Our main objective is to make a representation of its structure through co-citation. Also, the reviewer indicates questions that are far from this objective and that therefore cannot be answered at this point because we did not collect this type of information or fields (i.e. topics, popularity, editor, etc. ...). In addition, in line with our initial objectives, we opted to use Altmetric.com as our data source as it provides the sort of information that we needed and no other, i.e.: Mention Date (date of incorporation of the reference), Mention Title (title of the entry) and Mention URL (link to the revision of the entry in which the reference is introduced). Therefore many of the analyses indicated cannot be performed.  However, we do consider that the suggestions made by the reviewer can contribute to and improve the interpretation of the maps. That is why, first of all, we have conducted a more exacting statistical analysis in the following areas: 1) analysis of the annual evolution of citations by entry and of their accumulated value; 2) statistics concerning co-citations; and 3) a study of the multidisciplinarity of references.  In relation to averages and standard deviations, the reviewer is right and we expanded our explanation of this phenomenon. On the one hand, we have analyzed the Wikipedia entries that include more references, detecting, for example, those related to annual lists of scientific discoveries in a given discipline. On the other hand, we have also explained that this variation is not unknown in metrics of this type, since the distribution curve presents a particularly marked asymmetry as 81% of articles receive only one citation; those that receive between one and three citations account for 97% of the total. These are also seen in the fact that 20% of the most cited works constitute only 40% of the total number of citations. This phenomenon also occurs in almost all bibliometric indicators.  In this sense, following the reviewer's recommendations, we have carried out an analysis of the distribution of citations received by journals, which fits both log-normal and power-law. This explanation can be found, both in the results section and, in greater detail, in a new section that we have included in the discussion (Comparison of Wikipedia and Scopus). |
| **Scopus vs wikipedia**  (comment 1) The authors compare Scopus citations with Wikipedia citations in mean absolute difference. The total number of citations is likely different in Scopus and Wikipedia. If Scopus has significantly more total citations in its system, it is rather unsurprising most paper has more citations in Scopus than in Wikipedia. The hypothesis here can be "are the distribution of citations for the papers in Scopus significantly different from that in Wikipedia?" If you can reject the hypothesis, that gives a motivation for investigating what factors drive the tested difference in the distribution.  (comment 2) if there is a significant difference in the distribution of fields and the ranking of journal importance between Wikipedia and Scopus | This comparison was weakly explained in our first version of the paper and, therefore, we have edited it following the reviewers’ recommendation. Our aim was to determine whether the most cited works in Wikipedia coincided with those in Scopus. But obviously the work is not intended to analyze the citations or their distribution. However, we have followed the reviewer's advice and, as indicated in the previous reply, we have analyzed the distribution of citations in Wikipedia using the journals and comparing it with the results achieved in other works.  In addition to correcting this paragraph, we have exploited our data to improve the comparison between Wikipedia and Scopus citations at journal level in order to determine whether they offer different information. To do this we have used the number of citations included in the CiteScore Metrics (citations made in 2016 to articles published between 2013 and 2015) and adjusted those of Wikipedia to the same citation window. As we have commented in the text, the two statistical models generated offer an R2 of around 0.5 that we have not considered sufficient to demonstrate that there is strong relationship between both sets. |
| **Trolling**  Similarly, the finding that "General Medicine" and "Molecular Biology" stand out from other fields can be a result of the distribution of papers in each field. Imagine the editors are citing papers in random by throwing darts, trolling the system in some sense. | We thank the reviewer for pointing to the subject of trolling, which is one of the great problems of most social media, and we have incorporated the following paragraphs:  “The Wikipedia references, unlike those collected in other social media, offer remarkable quality control and transparency. In relation to the problem of trolling, the encyclopedia is based on a solid quality management system of post-publication peer review in which, in the case of discrepancies, changes are resolved through consensus between editors. Wikipedia also has two manuals: for non-academic experts (https://en.wikipedia.org/wiki/Help:Wikipedia_editing_for_non-academic_experts) and for researchers, scholars, and academics (https://en.wikipedia.org/wiki/Help:Wikipedia_editing_for_researchers,_scholars,_and_academics) both specifying the importance of the use of citations under the principles of verifiability and notability. This substantially minimizes the likelihood that references in entries will be tampered with.  Wikipedia also offers a complete list of its bots (https://en.wikipedia.org/wiki/Special:ListUsers/bot), including those such as the Citation bot (https://en.wikipedia.org/wiki/User:Citation_bot), which in addition to adding missing identifiers to references, corrects and completes them, something for which the digital encyclopedia offers several tools (https://en.wikipedia.org/wiki/Help:Citation_tools). However, this does not prevent the appearance of publications with a high, anomalous number of citations [42]. For instance, we found a report (https://www.altmetric.com/details/3171944) cited in 1450 lunar crater entries, not attributable to a bot. So, although the use of citations is not compromised, practices of this sort must be taken into account, for example, if their use is in an evaluative context. Given all of the aforementioned, we consider that in this context in which 193 802 Wikipedia entries and 847 512 article citations have been analyzed, it is very difficult to produce manipulations that could significantly alter the system and, consequently, the results achieved here.” |
| There needs t more evidence to support the main findings of this paper, namely from the **social perspective of Wikipedia**, the authors find a significative presence of “Medicine” and “Biochemistry, Genetics, and Molecular Biology” papers, **and that the most important journals are multidisciplinary**. One may find the same result from any perspectives, not exclusive to the social perspective of Wikipedia. There may not be anything particular about Wikipedia that associates with this observation. | We agree with the reviewer as long as, in principle, it may seem that there are similarities in the predominance of certain scientific areas (Medicine, ...) and certain types of journals (multidisciplinary). Although the reviewer indicates that the results are not exclusive to Wikipedia, we do consider that there are singular results in Wikipedia that are a direct consequence of the social practices of the platform. In this regard, we must indicate the specific case of "Biochemistry", which has a better representation—more acute in Wikipedia than in Scopus. The same happens with other areas such as "Agricultural and Biological Science", as observed in Figure 8. However, we do agree with the reviewer that these have not been properly described in the text. We have therefore improved the results and the discussion. We have emphasized the singularities of Figure 8, written two new paragraphs in the discussion, and added a new table as complementary material, which clarifies this and helps to better interpret the co-citation maps.  “Like other platforms, multidisciplinary journals are hubs and articulate the Wikipedia network. However, despite being a common global phenomenon, Wikipedia does have, and contains unique citation practices mentioning journals that are not cited or mentioned in a relevant way in other databases or platforms. This is evidenced in the scatter plot of Wikipedia and Scopus citations (see Fig 5).  This difference is also illustrated by a comparison of the journals most mentioned in Altmetric.com with those most mentioned in Wikipedia. As we can see, Wikipedia has the major multidisciplinary journals in common, as does Scopus. However, some of the most frequently mentioned journals in Wikipedia are the *Journal of Biological Chemistry* or *Zookeys* which are located in JCR’s Q2. Nonetheless, the *Journal of Biological Chemistry*, for example, is one of the most widely cited journals in the field of genetics receiving a large number of citations in various entries such as "Androgen receptor" (45 citations) or "Epidermal growth factor receptor" (25 citations). Therefore, Wikipedia points to another type of specialized journal in different fields (See Table S8) that are not identified in other rankings. In addition, as we have indicated, this can hardly be due to trolling or a bot.” |

**Comments of reviewer 2**

| **Comments** | **Response** |
| --- | --- |
| 1) This is study is directed related to the topic of creating scientific maps, a well-known research topic in the scientometric field. The authors should improve the section of related works, mentioning similar works. See and mention e.g. doi: 10.1016/j.joi.2016.03.008; doi: 10.1007/s11192-012-0784-8; The analysis of knowledge in information networks has also been conisdered in recent works:  doi: 10.1016/j.ins.2017.08.091 | We thank the reviewer for their suggestions. In addition to including these in our new text, we have improved our literature review by including nine more references:   - 1. Brzezinski M. Power laws in citation distributions: evidence from Scopus. Scientometrics [Internet]. 2015;103(1):213–28. Available from: https://doi.org/10.1007/s11192-014-1524-z - 2. Chang Y-W, Huang M-H, Lin C-W. Evolution of research subjects in library and information science based on keyword, bibliographical coupling, and co-citation analyses. Scientometrics [Internet]. 2015;105(3):2071–87. Available from: https://doi.org/10.1007/s11192-015-1762-8 - 3. Costas R, de Rijcke S, Marres N. Beyond the dependencies of altmetrics: Conceptualizing ‘heterogeneous couplings’ between social media and science. In: The 2017 Altmetrics Workshop [Internet]. 2017. Available from: http://altmetrics.org/wp-content/uploads/2017/09/altmetrics17_paper_4.pdf - 4. Didegah F, Thelwall M. Co-saved, co-tweeted, and co-cited networks. J Assoc Inf Sci Technol [Internet]. 2018 Aug 1;69(8):959–73. Available from: https://doi.org/10.1002/asi.24028 - 5. Seglen PO. Why the impact factor of journals should not be used for evaluating research. BMJ [Internet]. 1997 Feb 15;314(7079):498–502. Available from: https://www.ncbi.nlm.nih.gov/pubmed/9056804 - 6. Torres-Salinas D, Gorraiz J, Robinson-Garcia N. The insoluble problems of books: what does Altmetric.com have to offer? Aslib J Inf Manag. 2018;70(6):691–707. - 7. Trujillo CM, Long TM. Document co-citation analysis to enhance transdisciplinary research. Sci Adv [Internet]. 2018 Jan 1;4(1):e1701130. Available from: http://advances.sciencemag.org/content/4/1/e1701130.abstract - 8. van Eck NJ, Waltman L. Visualizing Bibliometric Networks BT - Measuring Scholarly Impact: Methods and Practice. In: Ding Y, Rousseau R, Wolfram D, editors. Cham: Springer International Publishing; 2014. p. 285–320. Available from: https://doi.org/10.1007/978-3-319-10377-8_13 - 9. Yan E, Ding Y. Scholarly network similarities: How bibliographic coupling networks, citation networks, cocitation networks, topical networks, coauthorship networks, and coword networks relate to each other. J Am Soc Inf Sci Technol [Internet]. 2012 Jul 1;63(7):1313–26. Available from: https://doi.org/10.1002/asi.22680 |
| 2) The authors should better explain why not using other types of networks to create the study. What is the effect of just using co-citation networks. Several options exists: citation networks, co-references, no use of references at all (e.g. text analysis). This possibility should be at least considered. | The reviewer is right and in the methodology section we have included a new paragraph in which we explain and justify the selection of the co-citation networks.  “Co-citation networks, bibliographic coupling and direct citations are some of the most significant bibliometric networks we can use to map citations from Wikipedia entries; of these, co-citation networks are the most popular in research [29,30]. If we take into account other types of network such as co-author and co-word, the aforementioned three methods show a high degree of similarity [31]. Within the field of altmetrics, the concepts of co-citation and coupling have both been adapted [32], but co-citations offer more varied alternatives [33]. Furthermore, they are of special interest as they have been identified as capable of enhancing transdisciplinarity [34]. Hence, we have generated co-citation maps at the level of journal, field and main field.” |
| 3) Why the authors used a dataset that has not been updated? | In our case, apart from the fact that the data used is one year old, when we started working on this study the only dataset available with the information we needed to carry it out was the CiteScore Metrics.Furthermore, until then Elsevier had updated this set annually, but then stopped giving it continuity. |
| 4) The authors should better explain the particular choice for a minimum covering tree and particular centrality measurements. The motivation for using such features of complex netowrks is not clear. | We have updated the methodology to better explain the choice of the Pathfinder algorithm and have included in the results a comparison between the network with and without Pathfinder, also introducing a new figure.  “The Pathfinder algorithm [35] has been applied as a pruning method following a common configuration (r=∞, q=n-1) that reduces the networks to a minimum covering tree. This algorithm—successfully applied in the field of Library and Information Sciences [26, 36]—keeps only the strongest co-citation links between all pairs of nodes and offers a diaphanous view of large networks. Given the huge amount of co-citations, especially between journals, we use this technique to prune them in order to make the networks more explanatory.” |
| 5) It is also not clear how interdisciplinarity (symmetry) is measured in graphs. It would be interesting the authors to mention some of the possible approaches in the literature and how  such methods could obtain different results. See and mention e.g. the follwoing examples: entropic diversity (doi: 10.1209/0295-5075/110/68001); quantum walks (doi: 10.1103/PhysRevE.88.032806) and concentric rings. | We appreciate the suggestion of the reviewer, but within the analysis of networks we have not analyzed the symmetry distribution. Our objective has been to map co-occurrences at different levels (journal, main field and field) established on the basis of citations from scientific articles. This is why we have decided to use the Pathfinder algorithm to highlight the strongest relationships. |
