# Supplementary material for: Science through Wikipedia: A novel representation of open knowledge through co-citation networks
Source: PLoS One. 2020 Feb 10;15(2):e0228713. doi: 10.1371/journal.pone.0228713 (PMC7010282; doi:10.1371/journal.pone.0228713)
Supplement: Attachment — Submitted filename: Response.docx [file pone.0228713.s013.docx]

# Response to the revision of the manuscript: “Science through Wikipedia: a novel representation of open knowledge through co-citation networks”

We greatly appreciate the review of the paper by the reviewers. We have revised it based on their very helpful comments. Below are the responses to reviewers’ comments.

**Comments of revisor 1**

| **Comments** | **Response** |
| --- | --- |
| With the additional statistical analysis, I believe the authors have done a good job mapping science on Wikipedia. However, I would like to suggest improvement about Table S1. Table S1 should include either mean/var of log-normal fit or exponent of power-law fit, with likelihood-ratio statistics of power-law model compared to log-normal model. Please be referred to  Clauset, Aaron, Cosma Rohilla Shalizi, and Mark EJ Newman. "Power-law distributions in empirical data." SIAM review 51.4 (2009): 661-703. pdf: https://arxiv.org/abs/0706.1062  for the rationales and methods for doing so. They also provide packages in R and Python. | We appreciate the reviewer’s suggerence but we have not carried out our power-law analysis by fields as suggested to improve Table S1. We used journals’ citations to illustrate this phenomenon in Wikipedia without dividing them by areas or papers (each journal is one observation). However, the recommended R package is the same that we used in this analysis which now has been referenced.  29. Gillespie CS. Fitting Heavy Tailed Distributions: The poweRlaw Package. J Stat Softw [Internet]. 2015 Mar 20;64(2). Available from: http://doi.org/10.18637/jss.v064.i02 |
| This leads to suggestions about Figure 5. Since both distributions are skewed, R2 is not an appropriate. Pearson R assumes finite variances, which sometimes breaks down when variables are power-law distributed. While I believe the conclusion that the correlation is not large, it is important to use appropriate method to reach this conclusion in the paper. I suggest the following two approaches:  1. Use rank correlation, such as Spearman's Rho or Kendall's Tao.  2. Use QQ plot to support the finding of outliers. Quantiles (p-value) at each axis should be computed using the power-law/log-normal models fitted for Scopus citation distribution and Wikipedia citation distribution.  I believe suggestion 2 above can replace Figure 5, which is currently too disperse. But if Figure 5 needs to stay for any reason, it should be plotted in log-log scale. Be careful to not fit linear models in log-scale, because variances do not remain constant in log-scale. One should fit the models before log-transform, and then visualize the fitted lines in log-scale. Suggestion 2 avoids all these complications. | Following the reviewer’s recommendations about Figure 5 we have updated it with colors by the ratio of Wikipedia and Scopus citation percentiles, and also included Q-Q plot using the normal and log-normal models and Figure 5 in log-log scale as supplementary material. These two new figures have been obtained taking into account the reviewer’s considerations. Furthermore, comments for both figures are included in results and discussion. |
| From suggestion 2, in fact, the authors can sort journals by the ratio of p-value of Wikipedia citation to that of Scopus under power-law models. The high-ratio journals are "over-cited" in Wikipedia, while the low-ratio journals are "under-cited", compared to Scopus as a baseline. I believe both the "over-cited" and "under-cited" ones worth some characterization and discussion. The authors may want to only look at journals with enough data to avoid noise in the ratio. Note that PLoS ONE and PNAS may not have the highest ratios, which suggests that the finding in the second paragraph of the result section may not be entirely correct. | We appreciate the recommendation to analyse the ratio of Wikipedia and Scopus citations under log-normal/power-law models. For this reason the over-cited and under-cited journals have been determined. To calculate them we filtered the dataset to only journals with a minimum of 2 papers and 3 Wikipedia and Scopus cites to avoid noise and then we calculated the ratio using their citation percentiles. As result we have included:  “In this sense we have obtained the journals’ citation percentiles in Wikipedia and Scopus, using only journals with a minimum of three citations in both platforms and two articles cited to avoid noise, and then the ratio between these percentiles have been calculated. While the commented journals have the same attention (ratio=1), the over-cited ones in Wikipedia are *Mammalian Species*(3559), *Art Journal* (192.56), *Northern History* (126.92), *European Journal of Taxonomy* (83.92) and *Art Bulletin* (80.92), and the under-cited ones are *Physical Review A - Atomic, Molecular, and Optical Physics*, *Dalton Transactions* and *Applied Surface Science* (all of them with 0.00027).” |
| This kind of comparison using ratio also applies in Figure 8. Looking at ratios, articles in the following fields gather more attentions in Wikipedia:  - Multidisciplinary  - Biochemistry, Genetics and Molecular Biology  - Agricultural and Biological Science  - Earth and Planetary Science  - Immunology and Microbiology  while the following fields are less popular in Wikipedia:  - Engineering  - Material Science  - Mathematics  - Chemistry  - Chemical Engineering  The less popular fields are all extremely hard, but steadily evolving sciences, which suggests that the limited ability of Wikipedia editors in comprehending articles in these fields restricted their ability to cite the articles. However, I have no empirical evidence nor reference to support this conjecture.  The field "Medicine" has roughly same percentages of attention in both Scopus and Wikipedia, despite the fact that it is the most popular field for both. This differs from the finding in the first paragraph of the result section. | The reviewer is right, the ratio analysis is useful to study the Wikipedia attention by fields. In this sense, the ratio of Wikipedia and Scopus citations would complete the results related to Figure 8. However, we have the limitation of Scopus citations, that only cover some years and papers, so we can not compare these results to Figure 8 properly. |
| As a side note, the authors also need to be careful when interpreting the correlation coefficients, which come with p-values. The size and significance of a correlation coefficient, while related, are not the same. The correlation can be small in size but statistically significant (e.g. huge sample and weak correlation), or large in size but not statistically significant (e.g. moderate correlation in a very small sample). For example, a correlation of 0.5, while statistically significant, means a sizable correlation. This correlation defines a pattern, then one can identify outlier points that do not obey this pattern. | In relation with the correlation coefficient comments we have reviewed them. Furthermore, these explications have been completed with the comments included about outliers. |
| Lastly, I would like to suggest the removal of the phrase "social perspective" throughout the paper. These is no analysis in this paper between the social aspect of Wikipedia editors and any of the finding. An example of such type of analysis can be the correlation of citation fields between editors who communicated in edit review, i.e. a social, communication network of editors. Merely co-edit of an entry does not imply any social interaction between the involved editors. While this paper is mapping science through the lens of Wikipedia, I have difficulty seeing how this mapping is done through social relations of editors. | We have changed “social perspective” to “Wikipedia perspective”. |
